# Depression, anxiety, and stress among university students in Selangor, Malaysia during COVID-19 pandemics and their associated factors

**DOI:** 10.1371/journal.pone.0280680

**Published:** 2023-01-25

**Authors:** Shun Sun Wong, Charng Choon Wong, Kwok Wen Ng, Mohammad F. Bostanudin, Suk Fei Tan

**Affiliations:** 1 School of Pharmacy, Management and Science University, Shah Alam, Selangor, Malaysia; 2 College of Pharmacy, Al Ain University, Abu Dhabi, United Arab Emirates; St John’s University, UNITED STATES

## Abstract

**Introduction:**

This study aims to assess the impacts of COVID-19 pandemics among university students in Malaysia, by identifying the prevalence of depression, anxiety and stress among them and their respective predictors.

**Methodology:**

An online cross-sectional study was conducted via non-probabilistic convenience sampling. Data were collected on sociodemographic characteristics, lifestyle, COVID-19 related influences. Mental health status was assessed with depression, anxiety, and stress scale (DASS-21).

**Results:**

388 students participated this study (72.4% female; 81.7% Bachelor’s student). The prevalence of moderate to severe depression, anxiety and stress among university students are 53.9%, 66.2% and 44.6%, respectively. Multivariable logistic regression analysis found that the odds of depression were lower among students who exercise at least 3 times per week (OR: 0.380, 95% CI: 0.203–0.711). The odd ratio of student who had no personal history of depression to had depression, anxiety and stress during this pandemic was also lower in comparison (OR: 0.489, 95% CI: 0.249–0.962; OR: 0.482, 95% CI: 0.241–0.963; OR: 0.252, 95% CI: 0.111–0.576). Surprisingly, students whose are currently pursuing Master study was associated with lower stress levels (OR: 0.188, 95% CI: 0.053–0.663). However, student who had poorer satisfaction of current learning experience were more likely to experience stress (OR: 1.644, 95% CI: 1.010–2.675).

**Limitations:**

It is impossible to establish causal relationships between variables on mental health outcomes, and there is a risk of information bias.

**Conclusion:**

The prevalence of mental health issues among university students is high. These findings present essential pieces of predictive information when promoting related awareness among them.

## 1. Introduction

Coronavirus disease, also known as COVID-19, is a highly contagious disease caused by a novel coronavirus known as the ’severe acute respiratory syndrome coronavirus 2’ or SARS-CoV-2. The WHO declared a global health emergency on January 30, 2020, and a global pandemic on March 11, 2020.

According to WHO [1], there have been 500,186,525 confirmed cases of COVID-19 and 6,190,349 deaths reported On 14 April 2022. Whereas according to the data from the Ministry of Health Malaysia, the total cumulative confirmed cases of COVID-19 have risen to 4,352,543 on 18 April 2022 in Malaysia [2]. Multiple studies have revealed the psychological impacts of COVID-19 pandemic towards various populations including but not limited to depression, anxiety and stress [3–8].

During the pandemic of COVID-19, significant moderate to severe psychological impacts, and moderate to severe anxiety among the China population in the early phase of the pandemic were reported in a study by Wang and colleagues [4]. Sharma and colleagues reported elevated stress and anxiety among Indian medical students [7]. Similar findings on prevalence of depression and anxiety [5], as well as higher levels of psychological distress were also reported among the general Malaysian population during the pandemic, psychological distress were significantly higher among those who were impacted financially by the pandemic and had higher levels of fear of COVID-19 [6].

Previous studies have shown that university students are more prone to have mental health issues [9–11] and are at higher risk of experiencing stress, anxiety and depression [12] as compared to general people [13]. It has also been reported that university students have poor mental including higher incidences of depression, anxiety, and high stress health in many countries including UK, US and Malaysia [14, 15]. Factors associated with poor mental health, particularly depression, anxiety and stress in university students, include sleeping patterns, financial, gender as female [15] as well as ethnicity as Chinese. In this study, we aim to assess the relationship of other factors such as the influence of the COVID-19 pandemic in Malaysia including the change in household income, having family members infected with COVID-19, influenced in social interactions, and having symptoms compatible with COVID-19 infections and change in mode of academic learning.

Although a systematic review concluded that screen time’s impact on mental health is small and negligible [16], Twenge and Campbell (2018) had suggested that younger adults with screen time usage of 4 hours or more were associated with lower psychological well-being and for those with 7 hours and above are 2 times more likely to ever have been diagnosed with depression and anxiety when compared to low or non-users of screen [17]. Therefore, the increased in screen time due to online classes and online academic activities during the pandemic and lockdowns can be a potential factor affecting mental wellness of university students.

There is limited knowledge about the depression, anxiety, and stress status of the COVID-19 pandemic among university students in Selangor, Malaysia. To have effective intervention in tackling mental health among university student, it is vital to first assess the mental health status of the students and to find out the vulnerable population. Therefore, this study was designed for the purpose: (1) to investigate the prevalence of depression, anxiety, and stress among university students in Selangor, and (2) to explore its association with sociodemographic factors and COVID-19-related influences, including financial, online education, and lifestyle. We hypothesize that the outcome of this study would provide essential pieces of information. Significant predictor that is highlighted in our study could help educators to target the vulnerable student population in planning effective interventions by targeting the right audience.

## 2. Methods

### 2.1. Study design and sampling criteria

An online cross-sectional study was conducted from May 7 to July 23, 2021, during and after the Phase 3 of Recovery Movement Control Order took place where students were gradually allowed to return to university students who are studying in universities located in Selangor, Malaysia were invited to participate in this online survey. Non-probabilistic convenience sampling was adopted where the data were collected anonymously through Google form which is written in English.

The online surveys were distributed to the target populations through social media, including Facebook groups, WhatsApp groups and Instagram. A question asking the location of their university was included to screen for participants studying in Selangor.

During recruitment, the inclusion criteria for potential participants are students studying in universities within Selangor. However, we excluded participants during data preparation if they were under 18 years old, above 35 years old or living outside the territory of Selangor. This data screening is done by asking respondents if they are students who currently lived in Selangor. Subsequently, they are also asked to fill in their age.

Selangor is a state on the west coast of Peninsular Malaysia, surrounding the capital Kuala Lumpur, Malaysia. It is a well-developed and progressive state in Malaysia. Being close proximity to Kuala Lumpur, Selangor has benefited from the booming economy and high-density population of KL city center. This also makes Selangor a logical destination for universities to effectively provide quality education for the betterment of future nation. According to the Department of Statistics Malaysia, Selangor contributes to highest percentage, 20.1% of Malaysian population in 2021 [18].

#### 2.1.1. Sample size

There are estimated 282,078 university students studying in Selangor [19]. With alpha value of 0.05, power of 0.95, proportion (p) of anxiety at 0.34, the sample size calculated is 344 [20, 21].

#### 2.1.2. Ethics statement

This study was approved by the Management and Science University Research Ethics Committee (MSU-RMC-02/FR01/09/L1/093). The design and process of the study were in accordance with the Declaration of Helsinki as revised 1989.

Information regarding the purpose and structure of this study was provided and written on the first page of the electronic questionnaire and were visible to the participant. Participants were informed that all the data obtained from the questionnaire was kept confidential and anonymous to protect the privacy of participants. They were also informed that these data were used solely for the purposes of this study only. Participation in this study is voluntarily basis. Online informed consent was obtained from all the participants of the study where participants need to tick a box to give their consent before they proceeded to next section to participate in this study.

### 2.2. Data collection

#### 2.2.1. Study variables

The adopted questionnaire consisted of questions covering several aspects of participant sociodemographic, lifestyle, COVID-19 related factors, and mental health status. Sociodemographic variables included age, gender, education level, marital status, type of university enrolled and household income. Information regarding participants’ lifestyle collected includes exercise, sleep pattern, personal and family history of depression, and time spent daily in front of screens. Moreover, participants were asked if they are having symptoms consistent with COVID-19, family members under the high-risk group of COVID-19, change in household income during COVID-19 pandemics, the current mode of learning, satisfaction of their current learning experience and their CGPA up to this semester. To prevent missing data, all questions in the survey were designed to be mandatory to be filled in before the participant can proceed to the next section. The questionnaire used for the study was pretested among 6 university students in Selangor, Malaysia, and changes were made in response to their feedback. Rather than keying the name of university. Respondents were asked to choose type of university (Private, Public, Others). In gender, “Diversity” option is replaced by “Others” in Gender.

#### 2.2.2. Outcome measures

For the evaluation of mental health status (Depression, anxiety and stress), the Depression, Anxiety and Stress Scale (DASS-21) were used as it is also used previously to evaluate the mental health of Malaysian students (20). It is a simplified form of DASS-43 which can measure all the psychometric properties to classify the stated of DAS [22]. DASS-21 consists of 3 subscales: Depression subscale, anxiety subscale and stress subscale, respectively. Each subscale consists of 7 items making it a total of 21 items. Each item scores from 0 (never), 1 (sometimes), 2 (often) to 4 (almost always). The depression subscale is constructed by items 3, 5, 10, 13, 16, 17, and 21 of the DASS-21. The total depression subscale score was interpreted as normal (0–9), mild (10–12), moderate (13–20), severe (21–27), and extremely severe (28–42). Items 2, 4, 7, 9, 15, 19, and 20 were used to assess the anxiety subscale score. The total anxiety subscale score was considered normal (0–6), mild (7–9), moderate (10–14), severe (15–19), and extremely severe (20–42). The seven items of the stress subscale are items 1, 6, 8, 11, 12, 14, and 18. The status of stress was considered as normal (0–10), mild (11–18), moderate (19–26), severe (27–34), and extremely severe (35–42) with total stress subscale score [23]. Questions under depression subscale measure the low self-esteem and poor outlook for the future. Questions in anxiety subscale measure fear response and physiological arousal while the stress subscale focuses on persistent arousal and tension [24]. The DASS-21 used in this study can be downloaded from: http://www2.psy.unsw.edu.au/groups/dass/. It is worthwhile to note that DASS-21 is only a screening tool to reflect the severity of symptoms, it cannot be used as a diagnostic tool.

#### 2.2.3 Covariates

Gender was divided into male and female categories. Educational levels were divided into three categories: Diploma or certificate, Bachelor’s or equivalent and Master’s or equivalent. Ethnicity was divided into four categories: Malay, Chinese, Indian and Others. Relationship status was categorised as single or in a relationship. The type of university was divided into public and private categories. Family income was categorised into B40, M40 and T20 [25]. B40 refers to income that is below that RM 4,360 while M40 refers to those whose household income that is between RM 4,361 and RM 9,619. Lastly, T20 refers to income that is above RM 9,620.

To study the indirect effect of pandemic and lockdown to the mental health of student, variables such as changes in income, lifestyle, health status and ways of learning were also included in our study. Changes of income affected by COVID was classified under increased, decreased and no changes. Exercise was classified as less than once a week, 1 to 2 times per week and 3 or more times per week. Sleep was categorised into normal (7–9 hours), more than normal (>9 hours) and less than normal (<7 hours). Participants were also asked if they are smoker or if they are second-hand smoker. On the other hand, the personal and family history of depression of participants were also recorded in this study.

They are also asked if their family are high risk group of COVID 19 (Yes or No). Since online study is partially implemented during the lockdown, students were also asked if they were having online or physical classes. Subsequently, they were asked if they were satisfied with their current learning experience (Yes or No).

### 2.3. Statistical analysis

Responses from 415 university students who are currently studying in any university located in Selangor were collected. Data were checked and cleaned to eliminate response that doesn’t fit the criteria, disengaged response and incomplete response. Only 388 respondents were entered and managed by Microsoft Excel; and analysed statistically with IBM SPSS Statistics for Windows, version 26.0 (IBM Corp., Armonk, NY, USA). Categorical data were reported as frequencies and percentages whereas continuous variables were summarised using mean and standard deviation. Bivariate analysis of categorical variables was conducted using Chi-square test (Fisher’s exact test if necessary) and Mann-Whitney U test.

The variable which had a significant association in bivariate analysis were then included in a multivariable logistic regression with a confidence interval of 95% to estimate the odds ratio (OR) and identify potential covariates associated with depression, anxiety and stress. The DASS scores are further divided into two categories: 1 = mild/moderate/severe/extremely severe and 0 = normal. Variables with a p-value of less than 0.05 were considered statistically significant in this adjusted analysis. The odds ratio and 95% confidence interval were reported from the multivariable model. Data were entered and managed by Microsoft Excel and analysed with IBM SPSS Version 26.

## 3. Results

### 3.1. General characteristics

Table 1 shows the findings of descriptive analysis of the selected variables of the university students in Selangor, Malaysia. A total of 388 university students had participated in this study; the average age of the students was 22.8 (SD ± 2.0) years. 72.4% of the participants were female, and 27.6% were male. Majority of the students were Chinese (41.5%) and Malay (41.3%), followed by Indians (13.4%), and 3.9% are from other ethnicities. Most of them are pursuing Bachelor’s or equivalent levels (87.1%), followed by Diploma/Certificate (13.4%) and Master’s or equivalent (4.9%). Eighty-four-point three percent of them are single, whereas only a minority of the participants are in a romantic relationship (15.7%).

**Table 1:** Descriptive statistic of all independent variables.

Variables	Frequency, *n*	Percentage, *%*	
***Gender***			
Female	281	72.4	
Male	107	27.6	
***Current Level of Study***			
Diploma/Certificate	52	13.4	
Bachelor’s or equivalent	317	81.7	
Master’s or equivalent	19	4.9	
***Ethnicity***			
Malay	160	41.2	
Chinese	161	41.5	
Indian	52	13.4	
Others	15	3.9	
***Marital Status***			
Single	327	84.3	
In a relationship	61	15.7	
***Type of University***			
Public	91	23.5	
Private	297	76.5	
***Family income***			
B40	198	51.0	
M40	144	37.1	
T20	46	11.9	
***Changes in household income in COVID-19 pandemic***			
Increased	23	5.9	
Decreased	201	51.8	
No change	164	42.3	
***Exercise***			
< 1 time/week	210	54.1	
1–2 times/week	120	30.9	
3 or more times/week	58	14.9	
***Sleep pattern***			
Normal (7–9 hours)	181	46.6	
More than normal (> 9 hours)	40	10.3	
Less than normal (< 7 hours)	167	43.0	
***Smoking***			
Yes	22	5.7	
No	321	82.7	
Second-hand smoke	45	11.6	
***Personal History of Depression***			
Yes	79	20.4	
No	309	79.6	
***Family History of Depression***			
Yes	45	11.6	
No	343	88.4	
***Are you having symptoms consistent with COVID-19*?**			
Yes	10	2.6	
No	378	97.4	
***Do you have family members that are under high risk group of COVID-19*?**			
Yes	134	34.5	
No	254	65.5	
***Current mode of study***			
Online	374	96.4	
Physical	14	3.6	
***Are you satisfied with your current learning experience*?**			
Yes	170	43.8	
No	218	56.2	
***CGPA up to this semester***			
2.0–2.99	61	15.7	
3.0–4.0	327	84.3	
***Score of DASS-21***			
	***Mean ± SD***		***95% CI***
Depression	16.10 *±* 11.60		14.94–17.26
Anxiety	15.20 *±* 10.83		14.11–16.28
Stress	17.89 *±* 10.88		16.80–18.97

Less than a quarter percent of participants is from public (23.5%) universities, and the rest are from private institutions (76.5%). Majority of the respondents reported their household income as B40, which is below RM 4,849 monthly income according to the Malaysia Income Classifications. At the same time, only 46 participants (11.7%) are from T20 families with household incomes above RM 10,960. This indicates that the participants are primarily from lower to middle-class families. More than half of the participants lead a sedentary lifestyle in which they exercise less than once a week; while only 14.9% of the respondent exercise 3 or more times a week.

More than half (53.3%) of the participants have abnormal sleep hours, 10.3% are more than normal (> 9 hours), whereas 43% have less than 7 hours of sleep per night. 20.4% of the participants reported to have previously been diagnosed with depression, and 11.4% have family history of depression. 10 participants (2.6%) reported symptoms consistent with COVID-19 infections, and more than one third (34.5%) of the participants have family members under the high-risk group of COVID-19. Large proportions of the respondents have online classes, and more than half of the respondents were unsatisfied with their current learning experience. Suha et al (2021) highlighted that poor interactivity and high complexity of the course could influenced the online learning engagement of students [26]. Tan et al (2022) and Azleen et al (2020) found that the learning experience, satisfaction and attitude of student in Selangor was average and the main challenge was the lacking of good and stable internet connection [27, 28].

### 3.2. Mental health

**Fig 1:**
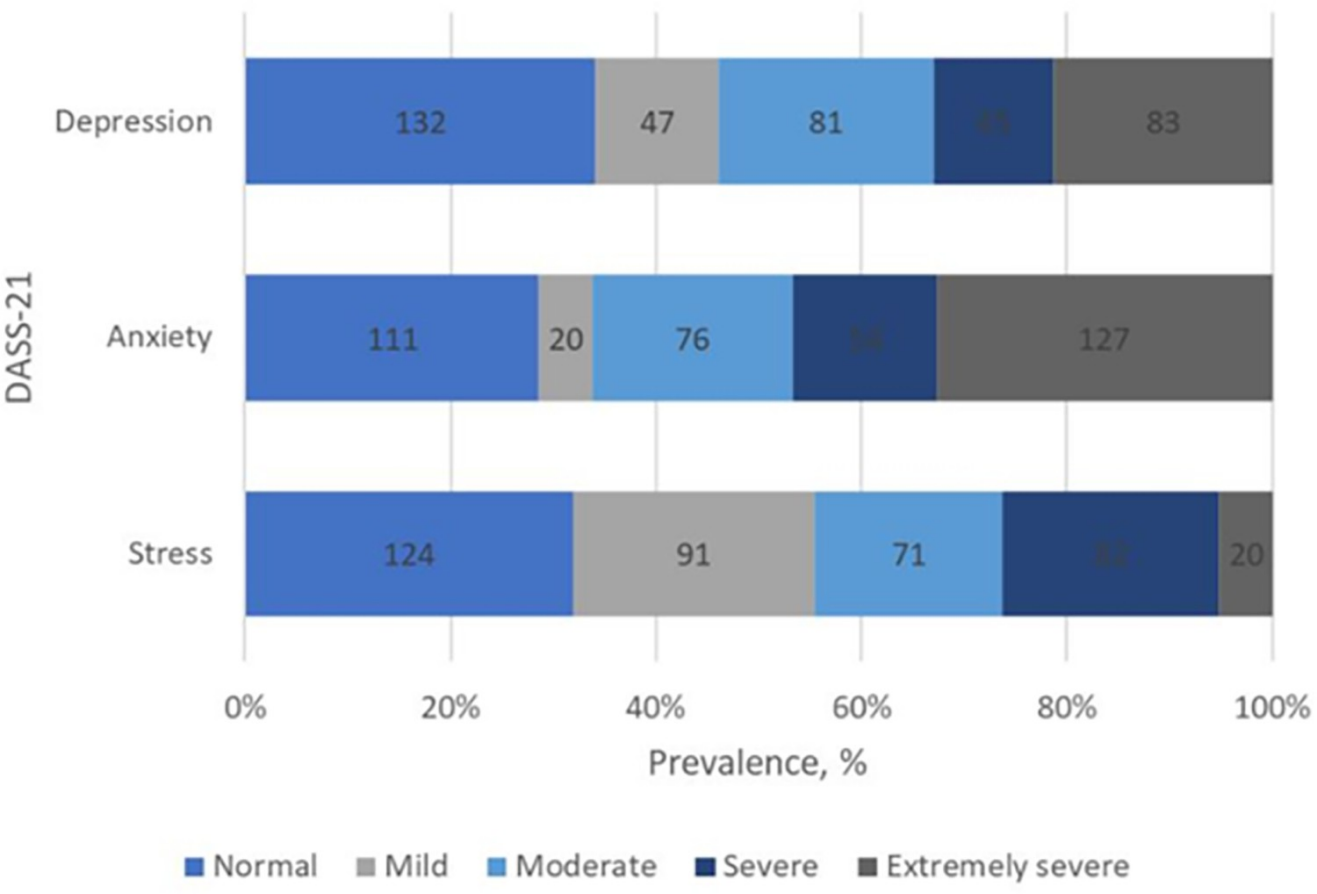
Prevalence of depression, anxiety and stress among university students studying in Selangor.

From the descriptive analysis of the 21-item Depression, Anxiety and Stress Scale (DASS-21), majority of the participants (34%) had normal scores on the Depression subscale, but 12.1% had scores in the mild range, and 21.4% is classified as severe. As for the anxiety subscale, about one-third of respondents (32.7%) was classified under extremely severe anxiety category in the anxiety subscale; fortunately, 28.6% and 5.2% of respondents had scored in the normal and mild ranges, respectively. On the other hand, 5.2% and 21.1% of respondents are classified under extremely severe and severe stress categories, respectively. Fortunately, majority of the remaining 32% of the participants scored normal in the stress subscales. The mental health of the participants from the results of DASS-21 subscales are summarised in Fig 1.

### 3.3. Significant predictors in multivariable logistic regression

As described in the Method of this manuscript, bivariate analysis was done to determine the variables that have a significant association with mental health outcomes. Variables that showed evidence of significant correlations were included in the multivariable logistic regression model. The model was then adjusted to achieved higher fitness (predicted with Nagelkerke R2) and significant predictors of our outcomes. Results of Chi-square and Mann-Whitney U analysis were shown in Table 2.

**Table 2:** Summary table of chi-square and mann-whitney U test findings.

			Depression		Anxiety		Stress	
			Normal	Depressed	Normal	Anxious	Normal	Stress
		N	N(%)					
Gender	Female	281	89 (31.7%)	192 (68.4%)	76 (27.1%)	205 (73%)	85 (30.3%)	196 (69.8%)
	Male	107	43 (40.2%)	64 (59.9%)	35 (32.8%)	72 (67.3%)	39 (36.5%)	68 (63.6%)
χ2 (p-value)			2.503 (0.114)		1.217 (0.270)		1.370 (0.242)	
Level_of_Study	Diploma/Certificate	52	18 (34.7%)	34 (65.4%)	16 (30.8%)	36 (69.3%)	14 (27%)	38 (73.1%)
	Bachelor’s or equivalent	317	103 (32.5%)	214 (67.6%)	85 (26.9%)	232 (73.2%)	97 (30.6%)	220 (69.5%)
	Master’s or equivalent	19	11 (57.9%)	8 (42.2%)	10 (52.7%)	9 (47.4%)	13 (68.5%)	6 (31.6%)
χ2 (p-value)			5.163 (0.076)		5.987 (0.050)*		12.492 (0.002)*	
Ethnicity	Malay	160	51 (31.9%)	109 (68.2%)	46 (28.8%)	114 (71.3%)	48 (30%)	112 (70%)
	Chinese	161	65 (40.4%)	96 (59.7%)	52 (32.3%)	109 (67.8%)	65 (40.4%)	96 (59.7%)
	Indian	52	15 (28.9%)	37 (71.2%)	11 (21.2%)	41 (78.9%)	11 (21.2%)	41 (78.9%)
	Others	15	1 (6.7%)	14 (93.4%)	2 (13.4%)	13 (86.7%)	0 (0%)	15 (100%)
χ2 (p-value)			8.843 (0.031)*		4.203 (0.240)		15.361 (0.002)*	
Marital status	Single	327	113 (34.6%)	214 (65.5%)	95 (29.1%)	232 (71%)	111 (34%)	216 (66.1%)
	In a relationship	61	19 (31.2%)	42 (68.9%)	16 (26.3%)	45 (73.8%)	13 (21.4%)	48 (78.7%)
	Married	0	0 (0%)	0 (0%)	0 (0%)	0(0%)	0 (0%)	0 (0%)
χ2 (p-value)			0.266 (0.606)		0.201 (0.654)		3.773 (0.052)	
Type of University	Public	91	33 (36.3%)	58 (63.8%)	32 (35.2%)	59 (64.9%)	32 (35.2%)	59 (64.9%)
	Private	297	99 (33.4%)	198 (66.7%)	79 (26.6%)	218 (73.5%)	92 (31%)	205 (69.1%)
χ2 (p-value)			0.266 (0.606)		2.502 (0.114)		0.562 (0.453)	
Family Income	B40	198	67 (33.9%)	131 (66.2%)	58 (29.3%)	140 (70.8%)	60 (30.4%)	138 (69.7%)
	M40	144	45 (31.3%)	99 (68.8%)	40 (27.8%)	104 (72.3%)	45 (31.3%)	99 (68.8%)
	T20	46	20 (43.5%)	26 (56.6%)	13 (28.3%)	33 (71.8%)	19 (41.4%)	27 (58.7%)
χ2 (p-value)			2.328 (0.312)		0.097 (0.953)		2.130 (0.345)	
Household income has ___________ after COVID-19.	Increased	23	11 (47.9%)	12 (52.2%)	6 (26.1%)	17 (74%)	10 (43.5%)	13 (56.6%)
	Decreased	201	61 (30.4%)	140 (69.7%)	57 (28.4%)	144 (71.7%)	59 (29.4%)	142 (70.7%)
	No change	164	60 (36.6%)	104 (63.5%)	48 (29.3%)	116 (70.8%)	55 (33.6%)	109 (66.5%)
χ2 (p-value)			3.641 (0.162)		0.113 (0.945)		2.219 (0.330)	
Exercise	< 1 time/week	210	57 (27.2%)	153 (72.9%)	53 (25.3%)	157 (74.8%)	57 (27.2%)	153 (72.9%)
	1–2 times/week	120	44 (36.7%)	76 (63.4%)	32 (26.7%)	88 (73.4%)	41 (34.2%)	79 (65.9%)
	> 2 times/week	58	31 (53.5%)	27 (46.6%)	26 (44.9%)	32 (55.2%)	26 (44.9%)	32 (55.2%)
χ2 (p-value)			14.552 (0.001)*		8.860 (0.012)*		6.926 (0.031)*	
Sleep	Normal	181	77 (42.6%)	104 (57.5%)	59 (32.6%)	122 (67.5%)	74 (40.9%)	107 (59.2%)
	More than normal	40	9 (22.5%)	31 (77.5%)	11 (27.5%)	29 (72.5%)	11 (27.5%)	29 (72.5%)
	Less tha normal	167	46 (27.6%)	121 (72.5%)	41 (24.6%)	126 (75.5%)	39 (23.4%)	128 (76.7%)
χ2 (p-value)			11.34 (0.003)*		2.780 (0.249)		12.684 (0.002)*	
Smoking	Yes	22	4 (18.2%)	18 (81.9%)	5 (22.8%)	17 (77.3%)	4 (18.2%)	18 (81.9%)
	No	321	114 (35.6%)	207 (64.5%)	96 (30%)	225 (70.1%)	109 (34%)	212 (66.1%)
	Second-hand smoke	45	14 (31.2%)	31 (68.9%)	10 (22.3%)	35 (77.8%)	11 (24.5%)	34 (75.6%)
χ2 (p-value)			2.947 (0.229)		1.536 (0.464)		3.678 (0.159)	
Personal History of Depression	Yes	79	14 (17.8%)	65 (82.3%)	12 (15.2%)	67 (84.9%)	8 (10.2%)	71 (89.9%)
	No	309	118 (38.2%)	191 (61.9%)	99 (32.1%)	210 (68%)	116 (37.6%)	193 (62.5%)
χ2 (p-value)			11.74 (0.001)*		8.745 (0.003)		21.744 (<0.001)*	
Family History of Depression	Yes	45	9 (20%)	36 (80%)	7 (15.6%)	38 (84.5%)	8 (17.8%)	37 (82.3%)
	No	343	123 (35.9%)	220 (64.2%)	104 (30.4%)	239 (69.7%)	116 (33.9%)	227 (66.2%)
χ2 (p-value)			4.458 (0.035)*		4.246 (0.039)*		4.708 (0.030)*	
Having symptoms consistent with COVID-19^a^	Yes	10	1 (10%)	9 (90%)	0 (0%)	10 (100%)	0 (0%)	10 (100%)
	No	378	131 (34.7%)	247 (65.4%)	111 (29.4%)	267 (70.7%)	124 (32.9%)	254 (67.2%)
χ2 (p-value)			2.639 (0.104)		4.113 (0.061)		4.821 (0.028)*	
Having high risk family member	Yes	134	44 (32.9%)	90 (67.2%)	31 (23.2%)	103 (76.9%)	40 (29.9%)	94 (70.2%)
	No	254	88 (34.7%)	166 (65.4%)	80 (31.5%)	174 (68.6%)	84 (33.1%)	170 (67%)
χ2 (p-value)			0.128 (0.721)		3.003 (0.083)		0.418 (0.518)	
Mode of Study^a^	Online	374	128 (34.3%)	246 (65.8%)	108 (28.9%)	266 (71.2%)	120 (32.1%)	254 (68%)
	Physical	14	4 (28.6%)	10 (71.5%)	3 (21.5%)	11 (78.6%)	4 (28.6%)	10 (71.5%)
χ2 (p-value)			0.192 (0.661)		0.367 (0.545)		0.077 (0.782)	
Satisfaction of Study Experience	Yes	170	69 (40.6%)	101 (59.5%)	55 (32.4%)	115 (67.7%)	68 (40%)	102 (60%)
	No	218	63 (28.9%)	155 (71.2%)	56 (25.7%)	162 (74.4%)	56 (25.7%)	162 (74.4%)
χ2 (p-value)			5.814 (0.016)*		2.077 (0.149)		8.997 (0.003)*	
CGPA	1.0–1.99	0	0 (0%)	0 (0%)	0 (0%)	0 (0%)	0(0%)	0 (0%)
	2.0–2.99	61	18 (29.6%)	43 (70.5%)	16 (26.3%)	45 (73.8%)	15 (24.6%)	46 (75.5%)
	3.0–4.0	327	114 (34.9%)	213 (65.2%)	95 (29.1%)	232 (71%)	109 (33.4%)	218 (66.7%)
χ2 (p-value)			0.657 (0.418)		0.201 (0.654)		1.807 (0.179)	
Sum of screen time	Median (IQR)		9.0 (7.0–11.0)	9.0 (7.0–12.0)	9.0 (7.0–10.0)	9.0 (7.0–12.0)	9.0 (7.0–11.0)	9.0 (7.0–12.0)
	U value (p-value)		14486.50 (0.020)^#^		12990.50 (0.015) ^#^		14545.50 (0.073)	

Fisher’s exact test is implemented when at least one expected cell count in a χ2 test of independence is <5

*Chi-square test (p< 0.05)

^#^Mann-Whitney U Test (p <0.05).

Table 3 depicts the association of selected variables with depression, anxiety and stress in a multivariable logistic regression model. Results of multivariable logistic regression indicated that exercise and personal history of depression are the significant predictors of depression. Student who exercised more than 3 times per week were less likely to be depressed (OR: 0.380, 95% CI: 0.203–0.711, p <0.01). On the other hand, On the other hand, the odd ratio of having depression was lower in who exercised 1 or 2 times per week compared to student who did not have exercise (OR: 0.723, 95% CI = 0.423–1.200. Similarly, student who had personal history of depression were less likely to be depressed as well (OR: 0.489, 95% CI: 0.249–0.962, p <0.05). 15.2% of the variances in the score of the depression subscales are explained by this model (Nagelkerke R2 = 0.152).

**Table 3:** Multivariable logistic regression analysis predicting depression, anxiety, and stress among university students who were studying in Selangor.

		Depression		Anxiety		Stress	
		OR	Sig.	OR	Sig.	OR	Sig.
Level of study							
	Diploma/Certificate	-	-		0.126	0	0.017*
	Bachelor’s or equivalent	-	-	1.203 (0.619–2.338)	0.586	0.835 (0.415–1.752)	0.664
	Master’s or equivalent	-	-	0.446 (0.146–1.359)	0.155	0.185 (0.053–0.649)	0.008*
Ethnicity							
	Malay		0.288				0.835
	Chinese	0.814 (0.494–1.34)	0.419	-	-	0.886 (0.520–1.504)	0.649
	Indian	1.045 (0.502–2.176)	0.905	-	-	1.287 (0.581–2.851)	0.534
	Others	5.691 (0.707–45.801)	0.102	-	-	5.242 (0.612–54.121)	0.990
Exercise							
	< 1 time per week		0.01*		0.021*		0.254
	1–2 times per week	0.723 (0.435–1.200)	0.209	0.957 (0.564–1.622)	0.869	0.794 (0.464–1.358)	0.399
	3 times or more per week	0.38 (0.203–0.711)	0.002*	0.425 (0.228–0.793)	0.007*	0.586 (0.307–1.119)	0.106
Sleep							
	Normal	-	0.147	-	-	-	0.194
	More than normal	2.119 (0.925–4.850)	0.076	-	-	1.253 (0.552–2.842)	0.589
	Less than normal	1.365 (0.835–2.231)	0.214	-	-	1.610 (0.961–2.696)	0.071
Personal History of Depression							
	Yes	-		-		-	
	No	0.489 (0.249–0.962)	0.038*	0.482 (0.241–0.963)	0.039*	0.245 (0.108–0.557)	0.001*
Family History of Depression							
	Yes	-		-		-	-
	No	0.585 (0.255–1.343)	0.206	0.587 (0.243–1.418)	0.236	0.713 (0.290–1.756)	0.462
Having symptoms consistent with COVID-19							
	Yes	-		-		-	
	No	-	-	-	-	0.001	0.999
Satisfaction of Current Learning Experience							
	Yes	-		-		-	
	No	1.392 (0.875–2.215)	0.163	-	-	1.6477 (1.033–2.723)	0.036*
Sum screen time of TV and other devices		1.096 (0.998–1.205)	0.056	1.113 (1.012–1.225)	0.028*	-	-
Nagelkerke R2		0.152		0.1		0.152	

* denotes p < 0.05

## 4. Discussion

### 4.1. Prevalence of depression, anxiety and stress

This study found that the prevalence of moderate to extremely severe depression, anxiety and stress among university students in Shah Alam are 53.9%, 66.2% and 44.6%, respectively. This is higher compared to some previous studies done among undergraduate students in Malaysia during the COVID-19 pandemic, whereby the prevalence for depression and anxiety were 29.4% and 51.3% respectively [29]. This might be due to the university regularly checked the mental health status of university students via regularly consultancy basis. Similarly, the prevalence of anxiety (55.1%) and stress (30.6%) a Malaysian study from August 4, 2020 to September 5, 2020 was also lower. However the prevalence of depression was slightly higher (59.2%) in their study [30]. This might be due to larger target population as the author gathered 1,163 participants across Malaysia. Another study done among university students in Malaysia by Islam et al (2018) reported the prevalence of depression was 29.4% before the pandemic [31]. In another study that uses Zung’s self-rating anxiety questionnaire to evaluate the extend of anxiety, the prevalence of minimal to moderate, marked to severe, and most extreme anxiety levels, were reported to be 20.4%, 6.6%, and 2.8% in a study that examined anxiety among university students in Malaysia during the COVID-19 pandemic [32]. In Rahman et al (2021) used the revised Impact of Event Scale (IES-R) to determine the impact of psychological impact of COVID-19 impact amongst the medical students in Sarawak, Malaysia, the prevalence of moderate to severe depression (16.7%), anxiety (16.9%) and stress (20%) was reported [33]. However, the proportion could not be compared as different tool were used in that study. Using DASS-42, Najlaa et al (2020) have found that 47.4% of the study population (pharmacy students) in Malaysia were having depression in their study [34]. In another study that uses, DASS-21, the author concluded that 65%, 67.21%, and 59.29% of the students reported having depressive, anxiety, and stress symptom during the lockdown period in Malaysia [35]. Variations could be due to differences in the study tools, university academic structure, geographical locations, and different years of data collection and other variabilities between studies. Interestingly, Raman et al (2019) found that 44% of a private university student had poor knowledge of mental health and only 21% of university student had good knowledge on that [36]. This would mean awareness campaign has to be carried out periodically to ensure university student has good understanding of the importance of mental health.

### 4.2. Significant predictors of depression, anxiety and stress

#### 4.2.1. Gender

In our study, bivariate analysis of gender and mental health outcomes does not show statistically significant association. This finding is inconsistent to Fawaz & Samaha (2020) study where they find females had significant higher stress compared to male with the findings of [37]. Although the prevalence of depression was higher among females (68.3%) than males (59.8%) in our study, being female does not mean they are more likely to be depressed. In contrast to our findings, a study conducted among public university students in Bangladesh had reported that female student in public university had higher prevalence of depression compared to male [38]. Our study also shows no statistically significant difference among gender in the prevalence of depression and anxiety. However, other studies conducted in Malaysia and China have observed significant differences in the prevalence of anxiety between genders [30, 32, 39, 40]. Females were reported in studies by Hou et al. (2020) and Simegn et al. (2021) to be more depressed, anxious and stressed [39, 40]. Kebede, Anbessie & Ayano (2019) and Sundarasen et al. (2020) also reported females to be more anxious than males [41].

#### 4.2.2. Exercise

In our study, the prevalence of depression and anxiety was higher among students who reported exercising less than 3 times per week than those who exercise 3 times or more per week. 59.8% of students who exercise less than 1 time per week and a relatively low percentage (10.5%) of students who exercise 3 times or more per week have depression scores in the DASS-21 within the ranges of mild to extremely severe depression. Whereas the reported prevalence of anxiety in students who exercise less than once per week, 1–2 times per week and 3 times or more per week are 56.7%, 31.8% and 11.6%, respectively. Past evidence suggests that physical inactivity is associated with higher levels of depressive symptoms. Physical exercises are deemed to have an antidepressant effect thereby is used as an alternative treatment for depressive disorders [41], and as adjunct treatment in anxiety disorders [42]. In earlier studies, it is concluded that physical exercises exert acute effects in reducing anxiety and depression [42]. This association is proven again in our study. Another study shows that the drive to exercise can be affected by lack of time (external barrier) and lack of motivation (internal barrier) [43]. As social interaction and the access to gym were limited due to MCO, student may also feel demotivated to exercise when there was limited resource and social support from peer.

#### 4.2.3. Personal history of depression

Students with personal history of depression have been shown to significantly have higher incidences of mild to extremely severe levels of depression, anxiety, and stress. This is consistent with the findings of previous studies, such as one conducted in France [42]. With this, we suggest the authority to have more interventions and resources to oversee students who have a personal history of depression to improve and maintain their mental wellness. This is crucial as an earlier study has shown about 30% of people who are depressed and acknowledged the need for treatment do not receive treatment as most of them either felt a financial burden, thought they could handle it without treatment (22.2%), not knowing where to go (16.7%) and fear of being committed or forced to take medications (15.2%) [44].

#### 4.2.4. Satisfaction of current learning experiences

Our study also found that students who were not satisfied with their current learning experiences were significantly associated with a higher prevalence of stress. Studies in Lebanon have also reported similar findings among university students during COVID-19 pandemics, where a significant relationship between satisfaction of online learning experiences and stress symptoms [44, 45] However, the satisfaction of current learning experience is not a significant predictor of depression and anxiety in our study. This finding is opposed to study done by Fawaz and Samaha (2020) [37]. Similar results of negative relationship between satisfaction of learning and psychological distress were also reported by a study conducted in Pakistan [37]. The difference in our findings could be explain by the geographical difference between studies, as study by Fawaz & Samaha (2020) was conducted in Lebanon, whereas our study was conducted in Malaysia [32]. Due to the geographical difference, there is difference in Internet speed, learning environment and other variabilities, where the definition and degrees of unsatisfaction in online learning experience varies. In addition, the variation could also be due to the fact that studies investigating the relationships of online learning experience satisfaction and its mental health impacts were limited.

#### 4.2.5. Sum screen time of the usage of TV and other devices

Our study finds that students who have higher sum screen time spent in front of televisions (TV) and other devices were 1.107 times more likely to experience higher levels of anxiety for everyone 1 hour unit increase of screen time (p<0.05). This association is also reported in Twenge & Campbell study (2018) conducted among adolescents. The study concluded that adolescents who had high screen time (7+h/day) were more than twice as likely to be ever diagnosed with depression and anxiety as compared to low users of screen (1 h/day) (17). A similar pattern was also reported in a study conducted among university students in the United States [46]. Screens of electronic devices emit blue lights with short wavelengths and can be detected by the suprachiasmatic nucleus in our brain, responsible for controlling the circadian rhythm [47]. Circadian rhythm is responsible to regulate our sleep at 24 hour-cycles by responding to light, to control sleepiness and alertness [48]. The body responses with delayed melatonin production, affecting the sleep cycles [49]. This can result in poor sleep quality and insomnia [50], and indirectly affects mental wellness.

### 4.3. Limitations

Although a robust and validated tool was used in our study, this study has several limitations. First, we are unable to establish any evidence for covariation between variable on the mental health outcomes (depression, anxiety and stress) as this was done cross-sectional rather than longitudinal. In addition, there could be some information bias due to the reliance on self-report by students. Due to limitation of movement, convenience sampling is employed instead of random sampling. As unstandardized coefficient is used in constructing the multivariable logistic regression model, we wouldn’t be able to compare which predictor variable has the greatest effect on the outcome.

## 5. Conclusion

A significant number of university students faced mental health issue during the pandemic. More than half of them suffered from mild to severe depression, anxiety and stress symptom during the period. Frequency of exercise, satisfaction on online learning, sum of screen time and personal history of depression were reported to be the significant predictor for depression, anxiety and stress in our study. These finding indicate the need for interventions that are more effective and more easily accessible to improve the mental health among university students even though we are now in endemic phase. These significant predictors in our studies should provides other researcher some clue in identifying the vulnerable population in online counselling programs in their interventional mental health program. Online education of mental wellness and regular screening should be done consistently via health awareness programs and incorporation into the academic programme structures as the mental health status among adolescent is knowingly high. Using these predictors, free online therapy sessions should be offered to high-risk groups which is identified during the screening process. As this was online self-assessment questionnaire, there was a lacking clinical assessment to confirm the presence of depression, anxiety, and stress. Further clinical assessment should be used to confirm the presence of such mental health disorders. Our study also highlights the needs of a large-scale longitudinal study for better understanding of the risk factors of depression, anxiety, and stress among university students in Malaysia at a larger scale.

## Supporting information

S1 File This is the SPSS raw data. (SAV)
